# THE OPPORTUNITIES AND CHALLENGES OF CONDUCTING OBSERVATIONAL RESEARCH IN LONG-TERM RESIDENTIAL CARE ENVIRONMENTS

**DOI:** 10.1093/geroni/igae098.3186

**Published:** 2024-12-31

**Authors:** Kirsty Haunch, Karen Spilsbury

**Affiliations:** University of Leeds, Leeds, England, United Kingdom; University of Leeds, Leeds, England, United Kingdom

## Abstract

Observational research has potential to offer new insights when addressing questions that matter for older people living in long term residential care settings (or care homes); moving beyond what people “say they do” to offer data of what is seen in every-day activities and interactions. However, there are challenges associated with why, how, where and in what context observations should be used in this setting. Care homes are dynamic care environments, and approximately 70% of older people living in care homes will live with dementia or cognitive impairment. These contextual and ‘people’ factors require careful consideration when choosing this method. Drawing on the experiences of two research teams, we share real world examples of the ethical, methodological, and practical concerns when using the observation method in the UK context. We offer examples from our work to guide researchers on: i) ‘making a case’ for observation; ii) the observational ‘gaze’ (or focus); iii) including people who lack capacity; iv) managing concerns about care or practices; v) the role of researcher; and vi) addressing observational sensitivities. As researchers, what we bring, what we do, and what we leave behind when using this method can have a significant impact on the lives (and work) of people in care homes. Our paper addresses a significant gap in the existing literature. It will be of interest to researchers world-wide and has the potential to benefit those living (and working) in care homes by enhancing the quality of research endeavours.

